# Time Distortions: A Systematic Review of Cases Characteristic of Alice in Wonderland Syndrome

**DOI:** 10.3389/fpsyt.2021.668633

**Published:** 2021-05-07

**Authors:** Jan Dirk Blom, Nutsa Nanuashvili, Flavie Waters

**Affiliations:** ^1^Outpatient Clinic for Uncommon Psychiatric Syndromes, Parnassia Psychiatric Institute, The Hague, Netherlands; ^2^Faculty of Social Sciences, Leiden University, Leiden, Netherlands; ^3^Department of Psychiatry, University of Groningen, Groningen, Netherlands; ^4^Amsterdam Brain and Cognition Center, University of Amsterdam, Amsterdam, Netherlands; ^5^Clinical Research Centre, Graylands Hospital, North Metro Health Service Mental Health, Perth, WA, Australia; ^6^School of Psychological Sciences, University of Western Australia, Perth, WA, Australia

**Keywords:** classification, multimodal distortion, protracted duration, psychological time, quick-motion phenomenon, slow-motion phenomenon, suprachiasmatic nucleus, Zeitraffer phenomenon

## Abstract

Of the perceptual distortions characteristic of Alice in Wonderland syndrome, substantial alterations in the immediate experience of time are probably the least known and the most fascinating. We reviewed original case reports to examine the phenomenology and associated pathology of these time distortions in this syndrome. A systematic search in PubMed, Ovid Medline, and the historical literature yielded 59 publications that described 168 people experiencing time distortions, including 84 detailed individual case reports. We distinguished five different types of time distortion. The most common category comprises slow-motion and quick-motion phenomena. In 39% of all cases, time distortions were unimodal in nature, while in 61% there was additional involvement of the visual (49%), kinaesthetic (18%), and auditory modalities (14%). In all, 40% of all time distortions described were bimodal in nature and 19% trimodal, with 1% involving four modalities. Underlying neurological mechanisms are varied and may be triggered by intoxications, infectious diseases, metabolic disorders, CNS lesions, paroxysmal neurological disorders, and psychiatric disorders. Bizarre sensations of time alteration—such as time going backwards or moving in circles—were mostly associated with psychosis. Pathophysiologically, mainly occipital areas appear to be involved, although the temporal network is widely disseminated, with separate component timing mechanisms not always functioning synchronously, thus occasionally creating temporal mismatches within and across sensory modalities (desynchronization). Based on our findings, we propose a classification of time distortions and formulate implications for research and clinical practice.

## Introduction

The way we experience time is a deep, hitherto unsolved mystery. Luminaries such as Edmund Husserl (1859-1939) and Martin Heidegger (1889-1976) who spent much of their prolific careers on trying to fathom the subjective experience of time, concluded that it is one of the most difficult of all phenomenological problems to understand (1, 2). Arguably, even more enigmatic are the experiences of time *distortion*, the topic of the present paper. With time distortion we mean a substantially altered perception of time in that the sense of time duration or the temporal relationship between events seems fundamentally altered. Thus, in their subjective experience, people perceive time as dragging along sluggishly, speeding up like in a Charlie Chaplin movie, or losing its chronological structure and assuming discontinuous, circular, or recurring patterns. The first medical descriptions of such distortions were published in the first decades of the twentieth century (3, 4), the same period in which Husserl and Heidegger worked out their philosophies of regular time perception. Both philosophers were probably oblivious to these publications or they would have mentioned them in their writings. Incidentally, they were not the only ones to overlook descriptive accounts on the topic; to this day there is preciously little knowledge about time distortions in spite of a blossoming research tradition in other aspects of time perception (5).

In the present context, the term *time perception* may need some clarification. It refers to a complex and multidimensional construct that enables us to make judgements regarding the duration and speed of unfolding of events, and their temporal ordering. Time perception is intrinsically linked to the sensory modalities. Traditional models of sense perception distinguish five sensory modalities (i.e., vision, audition, olfaction, taste, and touch), although it is possible to delineate 15 when other senses—such as the phenomenological sense of time—are included (6). In our overall perception of the world, time perception plays a fundamental role. Not only can events be perceived as proceeding more slowly or faster, time perception fulfills the metaphorical role of “conductor of the orchestra of the senses.” When our perception of time goes awry, so does our perception in other sensory modalities. As a consequence, our perception of time has important experiential and even existential ramifications. Our sense of “being-in-the-world” (7) depends on it, not only to gain a sense of constancy and continuity regarding events that unfold in time and space, but, crucially, also regarding our sense of self. After all, our sense of self-continuity is an essential constitutive condition to qualify as a person with a past and a future (8).

The term *time distortion* may also need some clarification. With it, we do not mean the mundane fluctuations in the perception of time that everyone experiences now and then. We have all experienced how time has a subjective, experiential aspect to it that may be out of tune with the fixed pace of clocks and other chronometers (9). Thus, time seems to drag when we are bored, while it seems to fly when we are engaged (10, 11), although, by the same token, a day spent in idleness may also seem to fly, just like an eventful 10 s may seem to last forever (12). However, these alterations are all within the spectrum of common everyday experience. With the term time distortion we do not even mean the more pronounced alterations that may occur in the context of conditions like mania or depression. Such instances of *tachypsychia* are typically in tune with the person's overall mental and physical state (13). Sacks (14–16), for instance, wrote numerous case vignettes in which tachypsychia plays a role, notably in mood disorder, Tourette's syndrome, autism, and the late stages of encephalitis lethargica, where lived time is substantially faster or slower when compared to physiological time indices, but nonetheless remains in accord with the patient's overall state.

In order to describe the temporal anomalies characteristic of Alice in Wonderland syndrome, the topic of the present paper, we define time distortions as time being out of tune with one's overall experience (17). Alice in Wonderland syndrome is the umbrella term for the spectrum of perceptual distortions that was first described by Todd (18). This “third group of perceptual disorders” (where hallucinations and illusions constitute the first two groups, see Table 1) comprises over 60 visual and other distortions that may occur individually or (less frequently) in combination with each other (20). The most commonly reported distortions are micro- and macropsia, where objects are perceived to be smaller or larger, and teleopsia, where objects appear to be farther away (21). The least reported, and probably the least known, are time distortions, exceptional phenomena that are best illustrated by an example. Ovsiew (22) described a 39-year-old, healthy man who, while taking a shower, saw “*drops seemingly hang in mid-air,”* the effect being “*very similar to the way bullets traveled in the Matrix movies, minus the trails that the effects crew added behind the bullets.”* This sensation of protracted duration lasted only minutes, but underlying it was an arteriovenous malformation that required neurosurgical intervention. Admittedly, this is a dramatic example, but one that aptly demonstrates how important it is to distinguish phenomena like these from tachypsychia and everyday experiences of altered time so familiar to most of us.

**Table 1:** Definitions of the three main classes of misperception [adapted from (19)].

Hallucination	A percept, experienced by a waking individual, in the absence of an appropriate stimulus from the outside world (e.g., seeing a cat that is not there, hearing a voice in the absence of sound waves)
Illusion	A percept, experienced by a waking individual, which is based on an appropriate stimulus from the outside world that is either misperceived or misinterpreted (e.g., taking a moving curtain for a cat or an intruder, hearing music in the monotonous drone of a computer fan)
Distortion	A percept, experienced by a waking individual, which is based on an appropriate stimulus from the outside world, of which, however, a highly specific aspect is altered in a consistent manner (e.g., seeing all straight lines as wavy, feeling one's head grow to an unnaturally large size, experiencing time duration or structure as altered)—collectively termed “Alice in Wonderland syndrome”

Time distortions are associated with numerous underlying conditions. They are perhaps best known in the context of substance use, but they also occur in neurological and psychiatric disorders such as stroke, head trauma, migraine, epilepsy, encephalitis, schizophrenia spectrum disorder, mood disorder, and catatonia (23–26). Regardless of their etiology though, they are classified as *perceptual distortions*, and therefore considered part of the Alice in Wonderland syndrome (13). As a consequence, an individual describing time distortions can be diagnosed as having Alice in Wonderland syndrome, even if it occurs in the context of another neurological or psychiatric disorder.

Phenomenologically, time distortions take various forms. Thus, time can be sensed as substantially accelerated or decelerated, sometimes to the point of complete stagnation. Apart from these quick-motion and slow-motion phenomena, time may also be experienced in even more bizarre ways, i.e., as going backwards, moving in circles, being fragmented, flowing together, or repeating itself (27). Finally, the ability to even sense the passage of time may be impaired.

Because studies of these temporal phenomena are few and a comprehensive overview is lacking, our goal here is to review the available literature on time distortions, where we define these as *substantial alterations in the immediate experience of time that are out of tune with the person's overall physical and mental state*, hoping to shed new light on their phenomenology, associated pathology, and etiology. Specifically, our research questions read as follows: (i) What are the demographic and clinical characteristics of individuals experiencing time distortions (whether formally diagnosed with Alice in Wonderland syndrome or not)? (ii) What types of time distortion are the most commonly reported? (iii) What sensory modalities are involved? (iv) What can the neurological and other clinical conditions associated with Alice in Wonderland syndrome tell us about underlying brain mechanisms? And (v), What is the clinical outcome of people dealing with time distortions?

## Methods

We carried out a systematic literature search in PubMed, PsycINFO, and Google Scholar up until February 1, 2021, using the search terms Alice in Wonderland syndrome OR time distortion OR temporal anomaly OR protracted duration OR quick-motion phenomenon OR slow-motion phenomenon OR Zeitraffer phenomenon, and supplemented the digital searches with backward searches. We included articles, books, and book chapters in English, German, French, Spanish, and Portuguese without date limits, provided they described original case reports or case series. Excluded were papers on related phenomena such as tachypsychia, time agnosia, disorientation for time, duration distortions, déjà phenomena, the loss of meaning of future time, time-gap experiences, and akinetopsia. The data extracted from each text comprised (i) year of publication, (ii) sex and age of the patient(s), (iii) phenomenological characteristics, (iv) clinical diagnoses, (v) test results, (vi) type of treatment, and (vii) outcome. To facilitate the interpretation of our findings, we newly devised the following classification of time distortions.

Type 1: Retrospective time-judgment errors
Type 2: Inability to correctly assess the passage of on-going time
Type 3: Inability to sense the passage of on-going time
Type 4: Quick-motion and slow-motion phenomena
Type 5: Bizarre alterations in the perception of time

## Results

**Figure 1:**
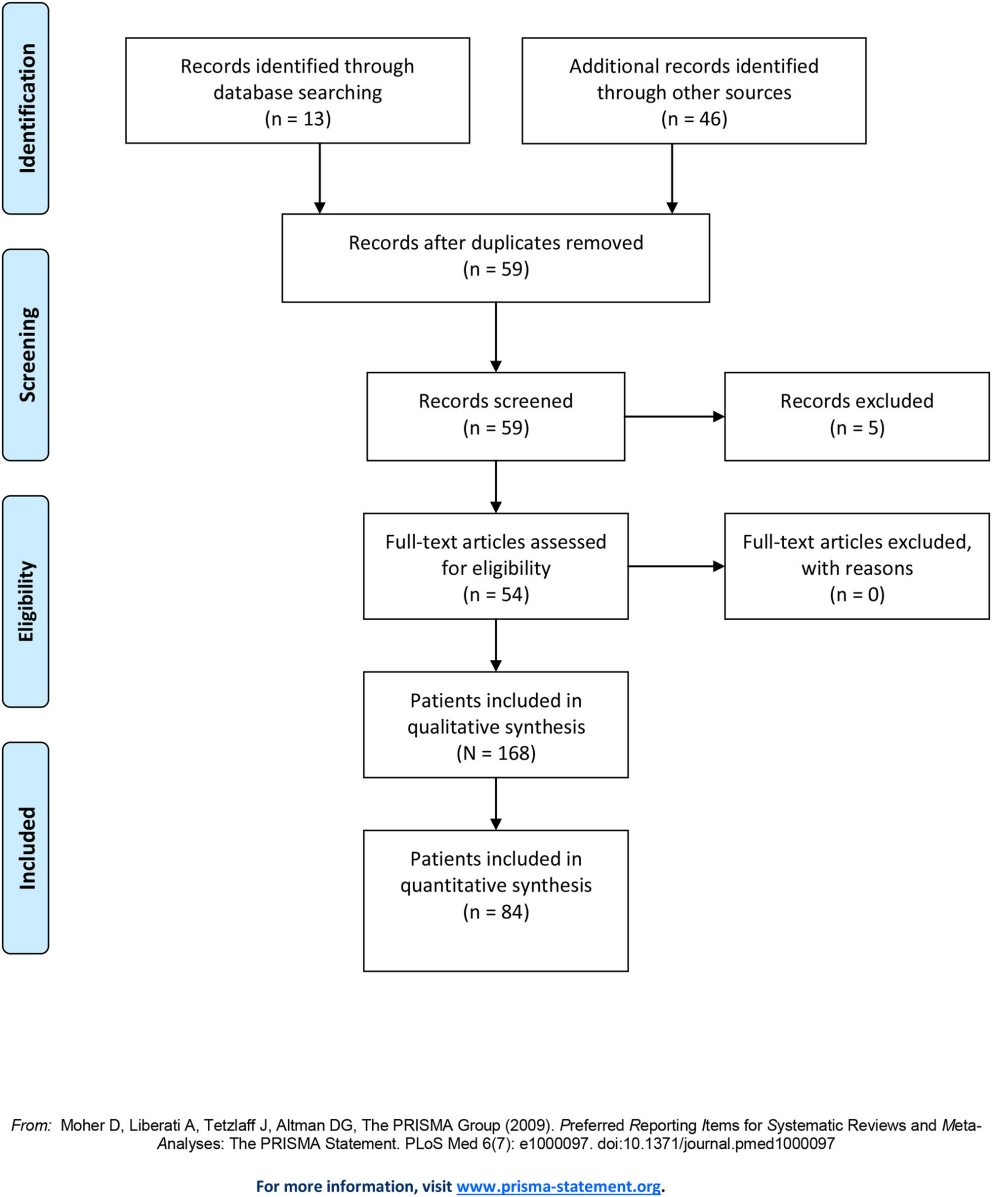
PRISMA flow diagram.

Our literature search initially yielded 13 papers on time distortions (for PRISMA flow diagram, see Figure 1). Cross-references and a search in historical sources yielded another 41 papers and five book chapters. Of these 59 texts, seven involved group-wise analyses of patient populations (total *n* = 85). Of these seven studies, three referred specifically to Alice in Wonderland syndrome (two in children, one in adults), while the other four described time distortions without mentioning a syndrome name. The remaining 52 studies provided detailed descriptions of individual cases and small case series (*n* = 89). These are summarized in Supplementary Table 1. Because five patients were described twice (3, 28–31) the number of unique case descriptions amounted to 84. Of note, one of these cases stemmed from one of the seven population studies, but since it was described in so much detail, we decided to include it in our analysis of individual cases. In total, we analyzed 168 cases.

### Population Studies (*n* = 85)

The seven population studies yielded data on time distortions as experienced by 85 individuals, of whom 21 were children. We summarized these as follows.

#### Adults, Post-surgery (n = 19)

The oldest population study on time distortions stems from Spiegel et al. (32), who investigated 30 adults who had undergone dorsolateral thalamotomy, a neurosurgical intervention for varying intractable conditions such as anxiety disorder, chorea, and central pain syndrome. Postoperatively, 19 of their patients experienced chronotaraxis, making gross judgment errors regarding their own age, the age of others, the time of day, the season and/or the year, sometimes also over- or underestimating the passage of time, to the extent that they appeared not to experience the passage of time at all (time distortion types 1, 2, and 3). These symptoms were independent of fluctuations of consciousness and typically lasted several days, although one patient took 6 months to recover from them.

#### Adults, Schizophrenia (n = 39)

Cutting and Silzer (33) conducted a study among 350 people diagnosed with schizophrenia, in which they described 45 cases dealing with the experience of time, six of which involved déjà phenomena. Unfortunately, the remaining 39 cases had such rudimentary descriptions that an allocation to types could not be made.

#### Children, Alice in Wonderland Syndrome (n = 10)

A third study, by Losada-Del Pozo et al. (34), included 20 pediatric patients with Alice in Wonderland syndrome of whom nine made mention of the speeding up of time. Since the cases were not described individually, it was unclear whether it involved type-2 or type-4 phenomena. Underlying medical conditions for the whole group comprised infectious disease (45%), migraine (40%), intoxication (10%), and epilepsy (5%). In a fourth study detailing nine boys diagnosed with Alice in Wonderland syndrome, Weidenfeld and Borusiak (35) described that one (included in Table 2) experienced brief, paroxysmal attacks of the quick-motion phenomenon (type 4). Although in the latter case epilepsy and encephalitis were ruled out, the underlying cause remained unknown.

**Table 2:** Conditions in the context of which time distortions are described in the literature, with numbers of patients mentioned in individual case descriptions (*N* = 84).

**Condition**	**No. of patients in case reports (%)**
**Intoxications**	**12 (14%)**
Cannabis	4 (5%)
Cardiazol	1 (1%)
Cocaine	2 (2%)
LSD	2 (2%)
Mescaline	1 (1%)
Phencyclidine	1 (1%)
Toluene	1 (1%)
**Infectious disease**	**4 (5%)**
Epstein Barr virus infection	2 (2%)
H1N1 influenza	1 (1%)
Parotitis	1 (1%)
**Metabolic**	**1 (1%)**
Insulin coma treatment	1 (1%)
**CNS lesions**	**26 (31%)**
Anti-NMDA receptor encephalitis	2 (2%)
Arteriovenous malformation	1 (1%)
Brain infarction	5 (6%)
Brain tumor	4 (5%)
Creutzfeldt-Jakob disease	1 (1%)
Encephalitis lethargica	1 (1%)
Head trauma	7 (8%)
Stroke	4 (5%)
Typhoid encephalopathy	1 (1%)
**Paroxysmal neurological disorders**	**25 (30%)**
Alice in Wonderland syndrome e.c.i.	3 (4%)
Cluster headache	1 (1%)
Epilepsy	8 (10%)
Migraine	12 (14%)
Oculogyric crisis	1 (1%)
**Psychiatric disorders**	**22 (26%)**
Bipolar disorder	1 (1%)
Depressive disorder	6 (7%)
Postpartum psychosis	1 (1%)
Psychotic disorder (schizophrenia)	14 (17%)

*Numbers do not add up to 100%, since several patients suffered from more than one condition*.

#### Adults, Migraine (n = 8)

The fifth study concerned a prospective cohort study on perceptual distortions in migraine conducted by Dooley et al. (36). At baseline, the group counted 95 patients, among whom seven (7%) reported quick-motion and slow-motion phenomena (type 4). Ten years on, three out of 60 patients (5%) reported these phenomena and 20 years on, this was eight out of the then remaining 28 (29%). The variation in these rates may be due to fluctuations in symptomatology and/or recall, but also, as indicated by the authors, to their omission to systematically inquire about time distortions early on in their study.

#### Children, Migraine (n = 8)

A smaller study by Smith et al. (37) included nine children with migraine, three of whom (33%) experienced time as passing more slowly and five (55%) as going faster. The study did not provide further specifications, barring that brain MRI and EEG were normal for all children.

#### Adults, Alice in Wonderland Syndrome Due to Vestibular Migraine (n = 1)

The seventh and final study is a cross-sectional survey among 17 patients with Alice in Wonderland syndrome due to vestibular migraine, of whom Beh et al. (38) report that one had experienced a transient episode of the slow-motion phenomenon (type 4).

In sum, the seven studies described 19 adults with type-1, type-2, and/or type-3 time distortions, nine individuals (eight adults and a child) with probable type-4 distortions, 20 children with type-2 or type-4 distortions, and 37 adults with unspecified types of time distortion.

### Case Reports (*n* = 84, Including One From the Population Studies)

The case reports that we retrieved are summarized in Supplementary Table 1. With 63% of the 84 patients being male, the sex ratio was 1.7:1. Age was known in 79% of the cases, with women having a mean age of 31 and men of 36 years (range: 6–68 years). Of all patients, 84% reported a single type of time distortion, 15% two types, and 1% three types. From these 84 patients, data on 100 time distortions were derived. Of the distortions reported, 3% were of an unspecified type. With 51%, type-4 phenomena were the most prevalent, of which 58% were quick-motion and 33% slow-motion phenomena, while 9% of the patients experienced both alternatingly. Next came type-2 distortions (inability to correctly assess the passage of time), which were reported by 29%, type-3 (inability to sense the passage of time) by 19%, type-5 (bizarre alterations) by 17%, and type-1 (retrospective time-judgment errors) by 2%. Noteworthy, the latter type-1 cases were accompanied by other types of time distortion.

#### Involvement of Sensory Modalities

**Figure 2:**
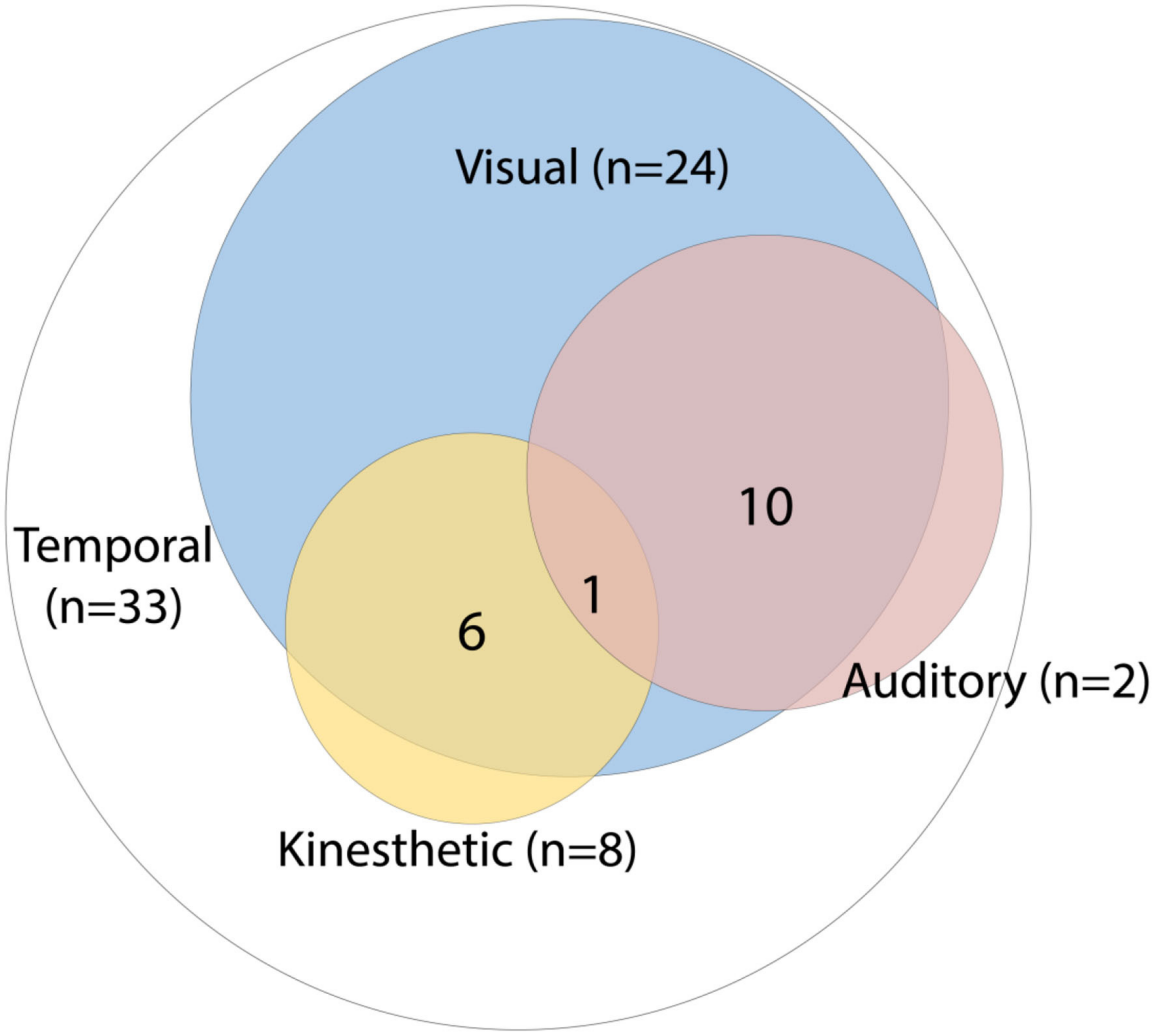
Distribution of sensory modalities involved in time distortions (*N* = 84).

The time distortions were limited to the sense of time passing in 39% of the 84 patients described. Since these experiences were not accompanied by reports of other perceptual changes, we designated them as unimodal in nature, meaning that only the phenomenological experience of the passing of time was affected. In 61% the time distortions were multimodal, with involvement of the visual modality in 49%, the kinaesthetic modality in 18%, and the auditory modality in 14% of these cases. Figure 2 illustrates the proportions, as well as the degree of overlap between the sensory modalities involved, with 40% being bimodal, 19% trimodal, and 1% involving four modalities. When the visual modality was implicated, people and objects in the environment were typically described as moving more slowly or faster, with patients often likening their experiences to cinematic effects. Six patients (7%) moreover reported involvement of the kinaesthetic modality, with three reporting an acceleration of their motor movements and three a deceleration. In addition to seeing things moving faster, eight patients (10%) experienced sounds as going faster as well, with five of them (63%) describing the sound as being of an unnaturally high pitch, as if a 33-rpm gramophone record were played at a speed of 45 rpm. One patient (1%) saw and heard things progressing more slowly, and another (1%) either faster or slower than normal. Two patients (2%) exclusively experienced time and speech as ensuing unnaturally fast, and eight others (10%) time and their own movements (kinaesthetic). The most complex case was described by Gloning and Weingarten (39), who portrayed a 49-year-old man with a brain tumor (probably thalamic) who saw things moving faster, perceived music and voices as going faster, and had the sense he himself was moving at high velocity, “*as if on roller skates.”*

#### Special Cases

Several cases that are of potential interest because of the various underlying brain mechanisms merit special mention. Three different papers report a *dissociation of the function of the central and peripheral fields of vision*. Hoff and Pötzl (29) were the first to describe how a single person could perceive both quick- and slow-motion phenomena simultaneously: their patient saw people and objects moving in slow motion in the central field of vision and zipping by in his peripheral field, describing how both phenomena were aggravated by loud noises, in the sense that things appeared to move even more slowly or faster when environmental sounds intensified. Becker and Sternbach (40) subsequently reported on a man who experienced people and objects in the central field of vision to be moving along rapidly but at a normal speed in his peripheral field. Gloning and Weingarten (39) described the opposite phenomenon of objects moving faster in the peripheral field and at a normal speed in the central field of vision. Another peculiarity of this latter case was that the patient experienced an aggravation of his symptoms when it became windy—curiously echoing an ancient hippocratic idea on environmental factors promoting epileptic seizures (41). Pötzl (42) writes of the ability to *abort* a quick-motion phenomenon in the case of a man who, during his epileptic aurae, saw “*flight lines*” that would “*pull him in,”* giving him the sensation of moving onwards with ever increasing speed. Pötzl goes on to report that this patient sometimes succeeded to stop the sensation by directing his gaze at the lower corners of a window, alternatingly to the left and to the right, thus “blocking” the flight-line sensation.

Another phenomenon worth singling out is a *temporal mismatch of different sensory modalities*. Levi and Miller (43) give the example of a man who experienced a “strobe light effect” that made him see moving people, objects, and scenes as a series of still pictures, and made him experience the movements of their mouths as out of tune with their voices. Likewise, our group described a woman who saw people's lips move discontinuously while hearing their speech without interruption (44). This had an alienating effect on her, comparable to watching a TV show with bad dubbing. Vatakis and Bakou (45) used the same metaphor to describe three cases they had collected from the literature (46–48) where sounds were out of sync with visually perceived movements, in the sense that mouth movements were perceived as lagging behind the words spoken (and in one case the patient's kinaesthetic sensation of his own mouth movements trailed behind the sound of his voice). The original reports preclude conclusions as to whether these are all modality-specific slow-motion phenomena or rather examples of differences in the *onset* of perception in different sensory modalities, as in the case described by Vatakis (48) i.e., cases of failed multimodal synchronization. We nonetheless mention them here because of their capacity to shed light on the brain's function in handling time differently within and across sensory modalities.

#### Diagnoses and Outcomes

In our series of 84 individual case reports, 95% contained information on associated clinical diagnoses. Although extremely heterogeneous, we divided these diagnoses into six groups: intoxications, infectious diseases, metabolic disorders, CNS lesions, paroxysmal neurological disorders, and psychiatric disorders (Table 2). Neurological disorders were diagnosed in 61% of the cases, psychiatric disorders in 26%. As to individual diagnoses, psychotic disorder (17%), migraine (14%), intoxication (14%), and epilepsy (10%) were the most frequent. Treatment was typically aimed at the underlying disorder, although some conditions were self-limiting (e.g., intoxications) or did not warrant therapeutic interventions for other reasons. Outcome was mentioned for 48% of the patients, of whom 68% attained full, and 8% partial recovery, where full recovery was defined as having fully recovered from the time distortion reported, not necessarily from the underlying condition. A quarter (26%) showed no recovery, while 13% (*n* = 5) of all cases ended in death, with causes comprising brain infarction, meningitis, encephalitis, Creutzfeldt-Jakob disease, and suicide. Although types 1–4 time distortions were seen in a broad range of different disorders, bizarre alterations in the perception of time (type 5) were an exception in the sense that they were seen mostly in the context of psychotic disorders (77%). Other contexts included encephalitis (*n* = 1), LSD use (*n* = 1), and an unknown condition (*n* = 1). Examples of these bizarre distortions were the sensation of time standing still, of living in an “*eternal now,”* of “*being thrown back in time,”* of “*dreaming backwards,”* and of “*time going backwards.”* Exemplary of the latter sensation was a patient who was convinced that the nurses managed to turn time back from 11:30 to 11:00 h, thus repeatedly forcing him to re-experience that same half hour.

#### Focal Brain Pathology

As to the localization of underlying brain disorders, 26 case descriptions provided some degree of information. Of these, 63% mentioned right-hemispheric lesions, 23% left-hemispheric lesions, 10% bilateral lesions, and 5% (*n* = 1) a thalamic lesion. Although the right hemisphere was thus involved in the majority of cases, further specifications were mostly unachievable because of the scattering of these lesions over occipital, parietal, temporal, and frontal regions (and the thalamus and pons). Neither were visual phenomena exclusively linked to occipital lesions or auditory ones to temporal lesions. In all, the most salient finding was the involvement of the occipital cortex in 53% of the right-sided and in 57% of the left-sided lesions.

## Discussion

Probably best known as one of the symptoms characteristic of Alice in Wonderland syndrome, we systematically reviewed 168 cases in which time distortions played a (major) role. Since the seven population studies we identified yielded only little information on individual cases, we will mainly be discussing the data we retrieved from the individual case reports (*n* = 84). We start with broadening the conceptual horizon, after which we, via physiological and pathophysiological considerations, will arrive at recommendations for clinical practice and future research.

### Historical Perspective

Even though lab-based timing experiments have been popular from the nineteenth century onwards, critical deviations in the subjective experience of time have long been neglected. As far as we know, they were first reported in early documents on substance use (9, 49, 50). Most of these reported on type-2 and type-3 time distortions. This is illustrated by Šerko (51), a physician who, during the early twentieth century, served as a test person at Kraepelin's clinic in experiments with the psychedelic hallucinogen mescaline. Having been injected with the substance, Šerko consistently overestimated the passage of time, thinking that an hour had passed when this was only 20 min. This got worse while the hallucinogenic effects kept increasing. As he elucidated in his self-report,

*At the height of the intoxication, the impairment of the sense of time is really huge. Especially when hallucinating abundantly, one has the feeling of swimming in a limitless river of time, somewhere and -when. One does no longer keep track of time, the immediate perception of time is deeply impaired. Again and again one has to make efforts to regain awareness of the temporal situation, to escape from this evaporation of time for a few moments*.

What Šerko here describes is the inability to properly assess the passage of time (type 2), followed by the inability—or at least a severely impaired ability—to experience time at all (type 3). In 1919, the Leipzig-based neurologist Heinrich Klien (1875-1941) was the first to publish the case of a time distortion observed in clinical practice. His article discussed an 8-year-old boy with probable epilepsy who experienced paroxysmal attacks during which he felt his body swell while seeing people moving about unnaturally fast. He also heard these people (and himself) speaking unnaturally fast [type-4 distortion with involvement of the temporal, visual, and auditory modalities (3)].

**Figure 3:**
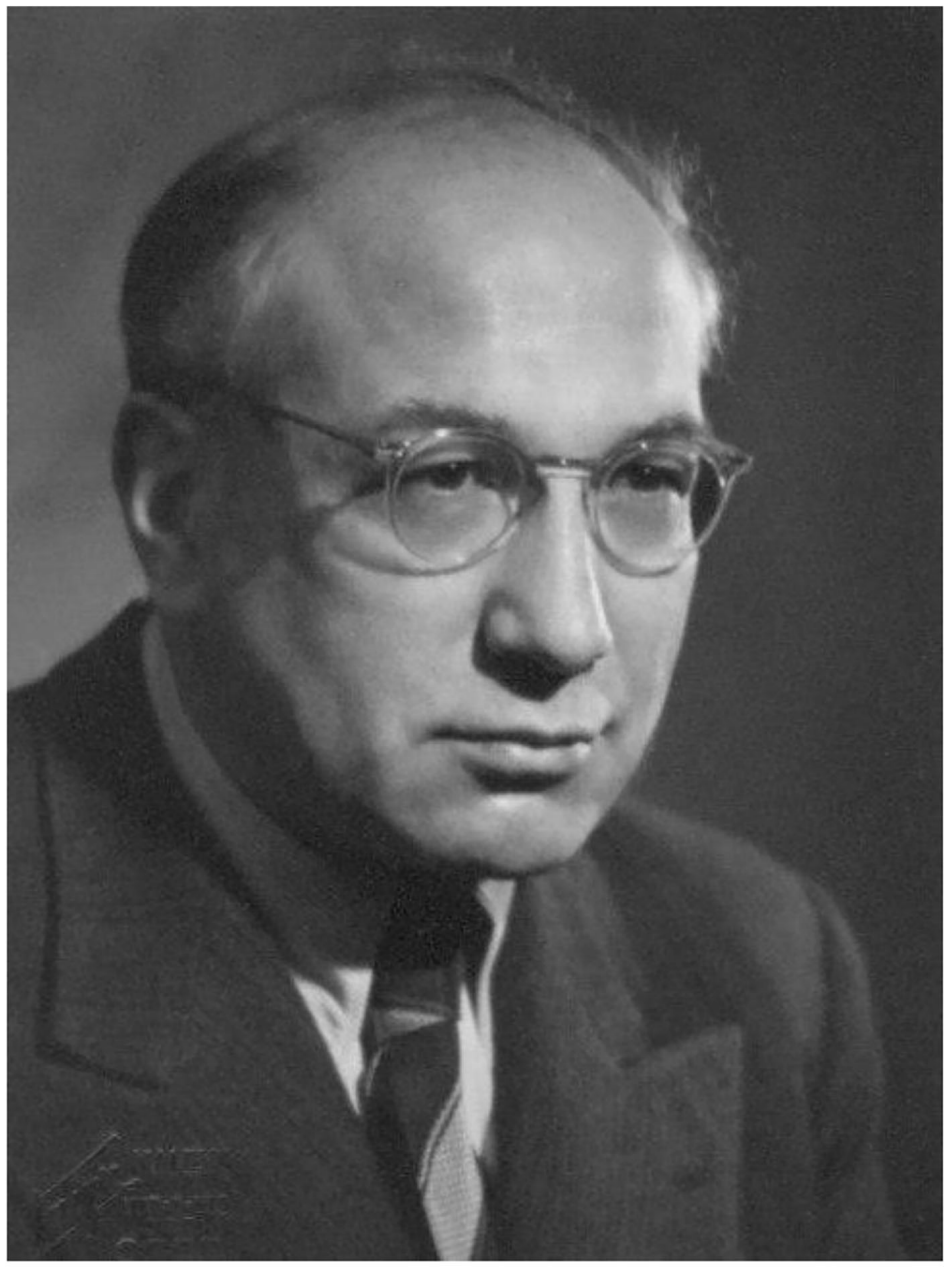
Hans Hoff (1897-1969), Austrian neurologist and psychiatrist. Source: Archives of the University of Vienna, 106.I.1075.

**Figure 4:**
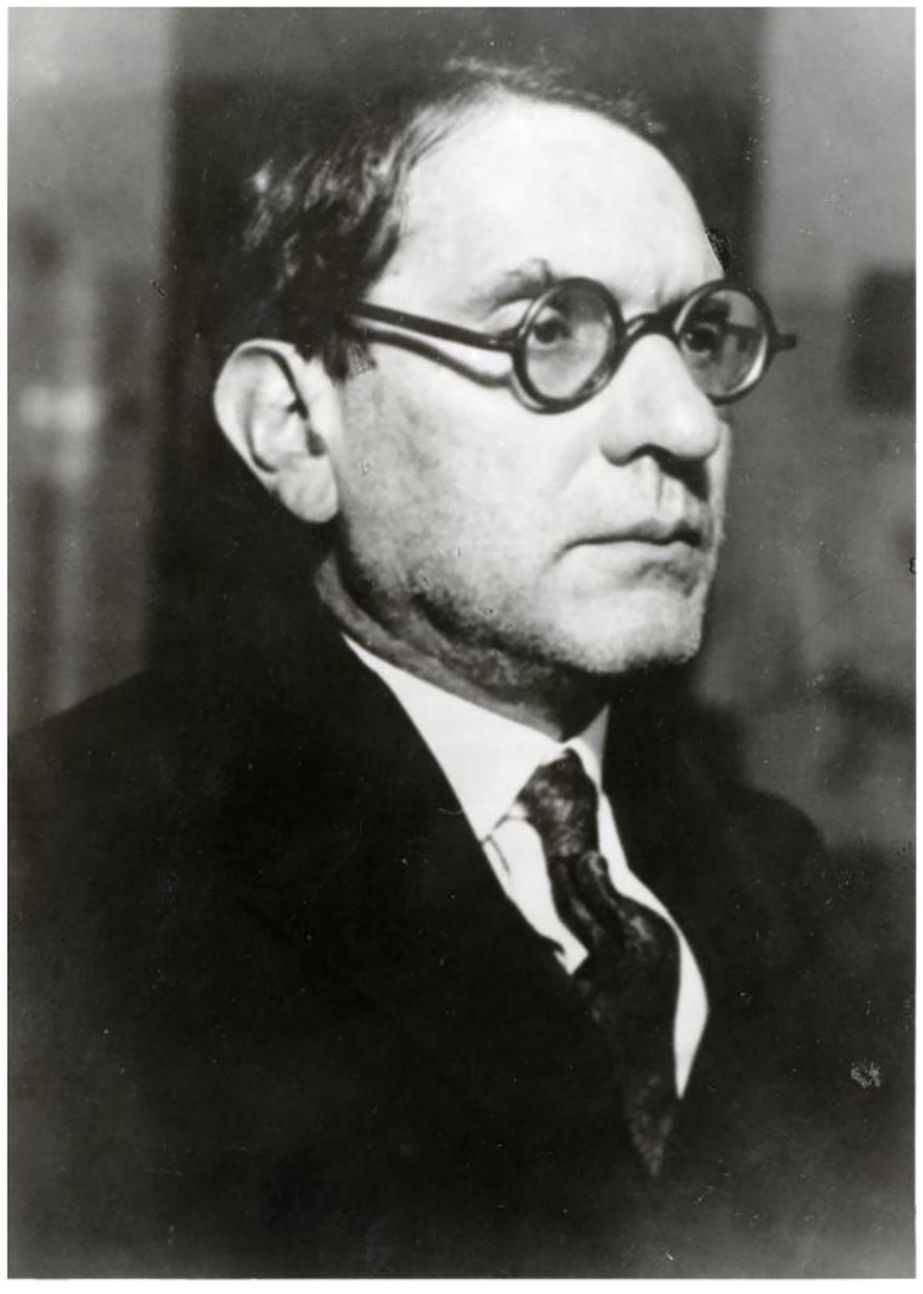
Otto Pötzl (1877-1962), Austrian neurologist and psychiatrist. Source: Image Archive of the Sigmund Freud Foundation, Vienna, B-699.

**Figure 5:**
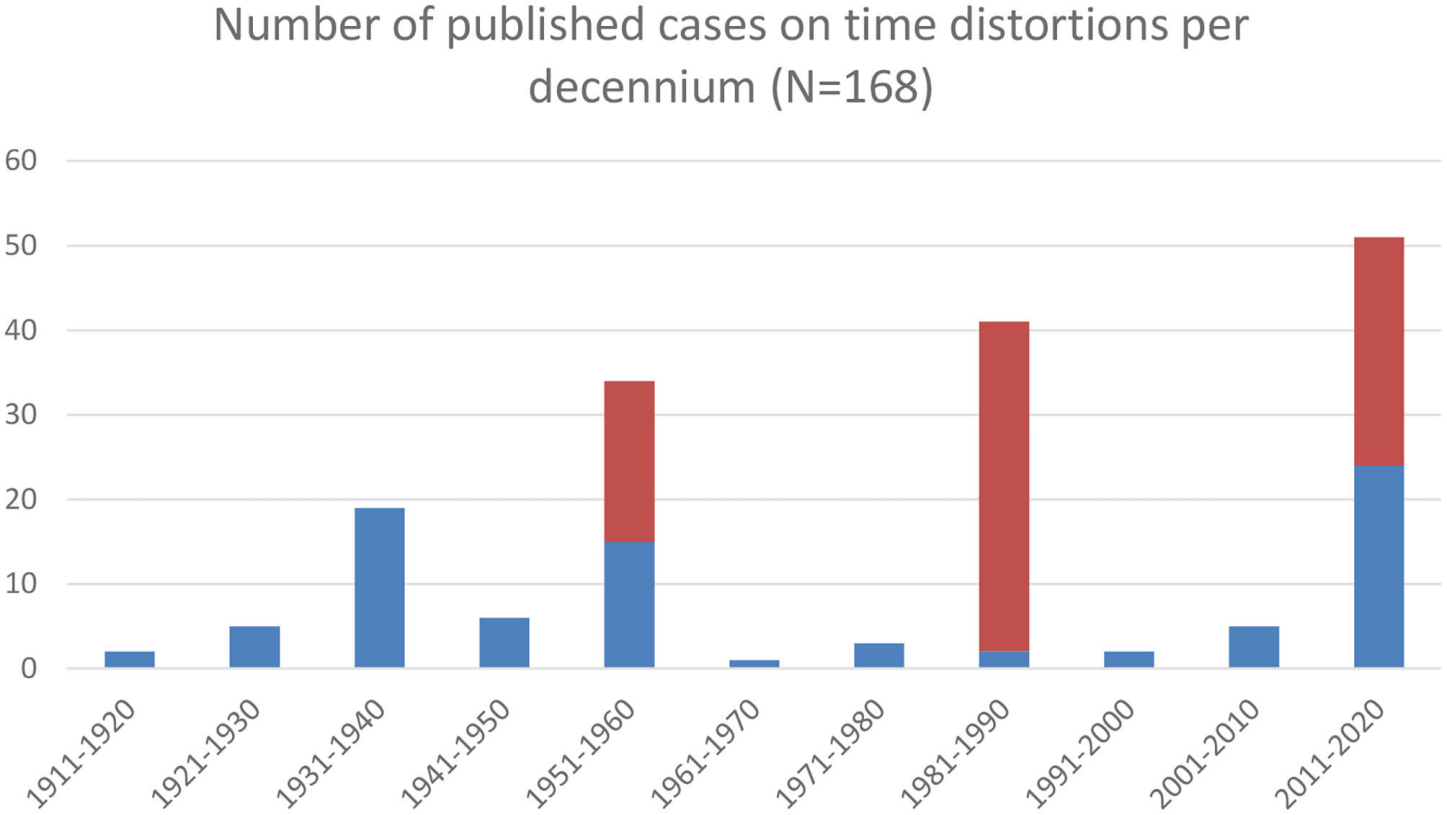
Number of published cases on time distortions per decennium. Blue = individual case descriptions; red = cases from population studies.

It was this observation that sparked the interest of Middle-European specialists such as Hans Hoff (1897-1969; Figure 3) and Otto Pötzl (1877-1962; Figure 4), who wrote several meticulous clinical case descriptions, some of which even contained autopsy findings. Their work allowed a first glimpse of the neural mechanisms underlying time distortions (to be discussed below). In 1934 they, moreover, introduced the term *Zeitrafferphänomen* (quick-motion phenomenon) for the condition previously described by Klien, as well as *Zeitlupenphänomen* (slow-motion phenomenon) for the opposite condition (28). More case reports in this vein were published around World War II, mostly in the context of traumatic head injuries, migraine, and epilepsy. Earlier though, especially Lewis (52) also described time distortions in psychosis. Thanks to his work, the 1930s saw a first modest peak in the number of case descriptions published (Figure 5). A second somewhat higher peak followed in the 1950s, with the highest but still modest peak occurring in the 1980s. As before, the already sparse interest in time distortions declined once more. It was not until the early twenty-first century that interest in these phenomena picked up again, with the number of published case reports rising to its current frequency of five per year—admittedly, still a very shy number. As a consequence, the true prevalance of time distortions is not known. Neither could we derive a comprehensive classification of time psychopathology despite several previous attempts (27, 33, 53, 54). Since none of these classifications covered the full spectrum of time distortions and related phenomena, we collapsed them into a single overarching classification, rendered in Table 3.

**Table 3:** Classification of time distortions and temporal anomalies.

**Time distortions**	**Description**	**Example**
Type 1	Retrospective time-judgment errors	Having the feeling that last year's summer was only yesterday
Type 2^*^	Inability to correctly assess the passage of on-going time	Grossly over- or underestimating durations
Type 3^*^	Inability to sense the passage of on-going time (“retention”)	Having no clue how much time has passed or how fast it is going
Type 4^*^	Quick-motion and slow-motion phenomena	Experiencing time as either accelerated or decelerated in the here-and-now, at odds with one's overall mental and physical state; sometimes in multiple sensory modalities
Type 5^*^	Bizarre alterations in the perception of time	Having the sensation that time is standing still, or moving in circles, or going backwards
**Temporal anomalies**		
Type 1	Disorientation for time	Not knowing what day it is, not knowing what time it is
Type 2	Time-gap experience	Having no memory of the previous stretch of time, as in hyperfocus or dissociation
Type 3	Tachypsychia	Experiencing time as either speeding up or slowing down, yet in accord with one's overall mental and physical state
Type 4	Temporal delusion	Being convinced that the past has never been, that the future will never come, that time is discontinuous, or that one has experienced the same situation before (déjà phenomena)
Type 5	Reduplication of time	Being convinced that one lives simultaneously in the present and in some other time
Type 6	Time agnosia	Being unable to grasp the concept of time and/or chronology
Type 7	Mystic time experience	Experiencing oneself as living in an eternal present

* *Characteristic of Alice in Wonderland syndrome*.

### Physiology

The neural mechanisms subserving human time experience rely upon the coordinated interaction of a large number of neuron populations that are distributed across specialist areas of the brain (55). These specialist areas, in turn, subserve different aspects such as duration evaluation, time expectation, temporal order, multimodal synchronization, and asynchrony detection. As summarized by Ovsiew (22), motion perception is subserved by cells in V5, while left or bilateral V5 plus the right posterior parietal cortex play a role in the temporal processing of visual stimuli. Whether all these interconnected structures project onto an “end organ” for time perception is as yet unknown (56, 57). A candidate structure for such an end organ was proposed by Craig (58), who tentatively identified the insula, with its function in integrating endogenous bodily signals, as a potential key player. Although as yet unproven, this proposal would make sense accepting that rhythmicity is a prerequisite for timing processes, and that numerous structures throughout the body have rhythms of their own, ranging from the suprachiasmatic nucleus (i.e., the “internal master clock,” which is involved in circadian rhythms) to individual cells (which may replicate at a rhythm of their own) and cell organelles (which may produce proteins, for example, at a rhythm of their own). The precise synchronization of all these internal rhythms is critical for an accurate experience of the flow of time—in the broadest sense—and perceptual awareness (59, 60). Their integration occurs in both local and central networks, in both parallel and sequential orders, and requires precise time-keeping systems. These latter are believed to require the use of multiple internal clocks, or pacemakers, whose role is to code temporal information and to orchestrate the integration of bottom-up and top-down processes (61, 62).

#### Scalar Timing Theory

Scalar timing theory (63, 64), an influential model for timing processes, states that the timing of intervals depends on pacemaker-generated pulses and on signals indicating the closing of the gates of an accumulator, thus facilitating the gathering of pulses in this accumulator. Together, the pacemaker, the pulses, the gate, and the accumulator constitute an “internal clock” (65). The number of accumulated pulses and the rate of their accumulation are translated into an estimated duration of the to-be-timed interval and transferred to working memory. The calculated duration is then compared to a similar time span from the reference memory. Finally, based on the comparison, an impression is gained about the duration of the event. Parietal and prefrontal cortices are thought to be involved in the indirect processing of time through attention and memory, and in the direct processing of time via cortico-striatal connectivity (66, 67). In addition to the internal master clock, widely distributed pacemaker systems are assumed to contribute to the subjective experience of the flow of time by accumulating temporal units over a given interval, resulting in a representation of the duration of time elapsed. A slowed pacemaker is then taken to result in a reduced accumulation of temporal units and thus in an underestimation of actual duration and vice-versa for a fast pacemaker (with time appearing to pass more slowly or faster, respectively) (55, 62). That such alterations may be more common than we tend to realize was shown in a cross-sectional study, in which Abe et al. (68) observed that no less than 3.8% of 3,224 healthy high-school students had experienced time as speeding up or slowing down.

#### Electrophysiology

Evidence for the role of central pacemakers comes from electrophysiological studies that show the synchronization of local oscillatory neural responses in cortical networks (69). Brain systems normally supporting temporal representations include the afore-mentioned internal master clock, the prefrontal cortex, posterior parietal cortex, thalamus, basal ganglia, cerebellum, and supplementary premotor areas (70–73). The prefrontal cortex plays a role through its capacity to represent the past, present, and future, and to integrate information across time (74). Together, the different areas involved constitute circuits that are linked to sensory motor processing via effector systems (69). Notably, frontoparietal systems also play important roles in attention and working memory, which are different from time-keeping processes but nonetheless interact with them in numerous ways.

#### Neurotransmission

At the level of neurotransmission, there is strong evidence linking timing mechanisms to dopamine (75). Since the interaction between frontal and mesolimbic regions and the role of basal ganglia in interval timing is mediated by dopaminergic connections (76), dopaminergic neurons in the basal ganglia have been proposed as the neural substrate of the internal clock. They are thought to facilitate the opening and closing of the gate of the accumulator, and to thus account for the rate of pulse accumulation (56). Studies show that dopamine manipulations can modulate time perception by altering the speed of the internal clock (62, 77, 78). For example, the administration of dopamine agonists and dopamine antagonists leads to an increase and a decrease of clock speed, respectively. Other neurotransmitters implied in timing mechanisms include glutamate, gamma amino butyric acid, and acetylcholine (79). Abnormalities of these neurobiological mechanisms have the potential to cause substantial changes in our sense of time (80), regarding duration (explicit timing) as well as temporal structure (simultaneity, order). Different mechanisms appear to play different (and sometimes highly specific) roles here. Thus, a recent study has indicated that dopamine has an important function in the processing of time duration, although not in discriminating the temporal order of visual stimuli (81). Sequence ordering of incoming sensations is required to generate an accurate understanding of the order of, and relationship between, events. Incorrect processing may therefore result in disruptions in the perceived flow of time and a decoupling of the so-called binding window of associability, the temporal period in which two stimuli can be experientially bound together and thus be sensed as occurring simultaneously or near-simultaneously (82, 83). This window is highly dependent on precise multisensory integration. Failure of the underlying mechanism may result in false action-sensation pairings. Thus, the awareness of touch may then lag behind the corresponding visual or auditory feedback, which is contrary to normal experiences where perceptual events are bound together and may lead to time flowing more uniformly (84). In the Results section, we detailed several examples of such temporal mismatches, where patients experienced spoken words as being out of sync with mouth movements.

### Pathophysiology

#### Lessons From Lesion Studies

On the basis of our findings and the theoretical framework elucidated above, we conclude that the network subserving our perception of time is widely disseminated. This is at odds with an earlier view held by authorities such as Hoff and Pötzl (28, 29), Critchley (85), and Cutting (54, 86), who all zoomed in on the right parietal lobe. Going by the then-available material, they linked quick-motion and slow-motion phenomena to right-sided brain lesions, notably those affecting right-parietal areas. Becchio and Bertone (87) likewise argued that the right hemisphere is crucial to our ability to perceive time, and that without it, time perception might be impossible. In a 15-year-old boy who experienced a transient slow-motion phenomenon, Kesserwani (23) found a circumscript brain infarction of the banks of the parieto-occipital area, which is part of the dorsolateral visual stream (the “where” pathway) that extends from V1 to the association cortices of area V6, and subserves the awareness of speed and direction. Because of the specificity of the lesion and the ensuing time distortion, the author concluded that this area must play a crucial role in our perception of time. The involvement of right parietal areas was also investigated with the aid of transcranial magnetic stimulation (TMS). By targeting either the left or right parietal cortex of six healthy volunteers for a duration of 10 min while having them carry out pitch and time discrimination tasks, Alexander et al. (70) tentatively identified the right inferior parietal lobe as necessary for the rapid discrimination of time intervals. Although their participants reported no symptoms reminiscent of time distortions, the authors nonetheless considered their findings to support Critchley's (85) observations regarding the role of the right parietal cortex in the quick-motion phenomenon. Despite several additional findings [e.g., (22)] that seemingly confirm this hypothesis, the present analysis indicates that time distortions—including type-4 phenomena—are also associated with right-sided frontal, temporal, and occipital areas, and even with some left-sided and mid-brain areas. Moreover, we established that the majority of cases (>50%) are associated with occipital lesions, and only less than half of that with right-sided parietal lesions (23%). This is consistent with the general model of physiological time perception summarized above, which indicates the involvement of a vast time-perception network of interrelated structures located throughout the brain, but also raises the important question of whether perhaps specific motion disturbances play a role in the cases we reviewed, which may or may not be integral to disturbances in time perception.

#### Lessons From the Dissociation of Central and Peripheral Vision

A tiny proportion of those reporting on time distortions described experiencing the very rare dissociation of the velocity of objects perceived in their central and peripheral fields of vision. This peculiar phenomenon suggests that different parts of the brain appreciate the speed of moving objects differently. The retinotopic representation of central and peripheral vision is such that the central visual field projects on occipital areas V1, V2, and V3, whereas the peripheral fields project on more anterior areas, along the medial wall of the occipital cortex, all the way across the fundus of the parieto-occipital sulcus (88). We already saw that the brain sometimes appears to process information in different sensory modalities in different ways, thus accounting for the occasional occurrence of a temporal mismatch between sensory modalities (“making” a person's lips to seemingly move out of sync with what is being said, for instance). Physiologically, there is a small time lag between visual information and auditory information, and information from these sensory modalities must therefore be recalibrated to become integrated into a synchronous, coherent whole. A likely explanation for multimodal desynchronization is therefore a difficulty in integrating that information, rather than a slowing down of one information channel. Apparently, something similar holds true for information within a single sensory modality (in this case the visual modality) when it is processed in separate areas, i.e., in visual areas V1-V3, vs. more anterior regions, the net result being an incongruity in the speed of visually perceived objects traversing one's field of vision.

#### Lessons From Clinical Studies

The large number of associated clinical conditions that emerged from our analysis underscores that the temporal network can be compromised by numerous aetiological agents. However, our finding that type-5 time distortions are linked predominantly to psychotic disorders suggests that an excess of postsynaptic dopamine transmission might be responsible for these bizarre phenomena. However, this is not supported by a recent study on the role of dopamine in time perception, where a depletion of dopamine precursors was found to affect duration processing but not the discrimination of the temporal order of visual stimuli in healthy volunteers (81). As a consequence, the dopamine hypothesis for these bizarre time distortions in psychosis is in need of further study. Alternatively, the bizarreness of the manifestations may be a reflection of the various formal thought disorders known to occur in psychosis. Since we can only rely on people's—by definition subjective—accounts of time distortions, especially in schizophrenia spectrum disorders it is important to distinguish between form and content. After all, in this group it is sometimes hard to tell whether patients provide descriptions of something they perceive or merely make cryptic statements that we should not attempt to connect with actual experiences. To illustrate this, we here offer an example from Fischer's (4) publication where a patient is quoted as stating that, “*this is not his wife but his spouse, that he has been at the hospital for 100 years, and that time is running out here as if oiled*.” In analogy with the work of Myers and Murphy (89), it would therefore seem preferable to label such unintelligible utterances as “reported temporal distortions” rather than taking them for accurate accounts of something actually perceived. Future studies might well benefit from attempts to disentangle such contradictions between reported time and experienced time (if possible at all).

#### A Central Role for Time Perception in Psychopathology?

Another lesson from clinical studies is that time distortions, and perhaps alterations in the perception of time in general, may play an as yet underestimated role in several types of psychopathology. It is no coincidence that Cutting and Silzer (33) spotlight the great Eugène Minkowski (1885-1972): “[Minkowski] *was so impressed with the alteration of time-sense in schizophrenia that he fashioned his entire theory of the condition around this single phenomenon. To him the most characteristic feature of schizophrenia was what he called “a lack of vital contact with reality,” in which an altered appreciation of time was the major component.”* Elaborating on this theme, Cutting and his group introduced aberrations of time perception as the core of various types of psychopathology. For example, based on a qualitative analysis of hundreds of narratives of decelerations of subjective time provided by patients with major depressive disorder, they reconceptualized depression as “*a disorder of lived time*” (90). They further argued that the other symptoms of depressive disorder are secondary to this characteristic slowing-down of subjective time. If anything, their work underscores the centrality of time to our experience of the world. Ultimately, personal time experience is a crucial aspect of the way we connect to our surroundings. It creates a structure whereby our experiences and feelings become integrated and continuous.

A fragmentation (rather than deceleration) of that continuity was related by patients diagnosed with schizophrenia, to whom life felt like a series of discrete snapshots rather than one continuous flow (91). This is in line with the curious time perceptions (type-5 distortions) reported by a subset of the cases we reviewed, which were chiefly associated with psychosis. In the light of these observations, it is tempting to speculate that fluctuations in subjective time make one lose connection with reality, up to the point of frank psychosis, whereas a deceleration of subjective time facilitates depression.

### Clinical Practice

#### Diagnosis

In clinical practice the identification of time distortions hinges on apposite history-taking and an adequate insight into the different phenomenological (sub)types. To link the ensuing findings to underlying pathology, a comprehensive psychiatric and neurological work-up are requisite, as well as blood work, an EEG, a brain MRI, and—on indication—a lumbar puncture, neuropsychological and toxicological evaluations, and/or a medication review. As alluded to above, the differential diagnosis of underlying conditions is broad, while from a phenomenological perspective there also are several conditions from which time distortions need to be distinguished.

#### Differential Diagnosis

The differential diagnosis of time distortions includes tachypsychia (changes in the apparent speed of time that are in tune with one's overall physical and mental state), time agnosia (a loss of comprehension of the succession and duration of events), disorientation for time, duration distortions, déjà phenomena (subjectively inappropriate impressions of familiarity of a present situation with an undefined past), the loss of meaning of future time, time-gap experiences, and akinetopsia. Especially the latter two may be difficult to tell apart from time distortions.

Time-gap experiences are characterized by the impaired recollection of a recent stretch of time such as the last hour of a long-distance journey (92). Often, the underlying problem is a hyperfocus on aspects that require one's attention (e.g., driving, reading, writing, gaming, fishing), an ensuing loss of awareness of one's surroundings, and hence a loss of the sense of time passing. These experiences also occur in the context of acute stress, dissociation, and epilepsy, but they are mostly physiological in nature and only rarely warrant medical attention.

Akinetopsia is the inability to see movement. We are accustomed to seeing objects move, but—although rare—it is possible to see the colors and shapes of objects without being capable of registering their movements. As several cases in our overview indicate, it may not always be easy to make a proper distinction between time distortions and akinetopsia. Hoff and Pötzl (29) for example, described quick- and slow-motion phenomena in a 62-year-old man who also saw people standing still and then popping up in different postures in different places, apparently unable to see them move there and instead merely seeing “snapshots” of their trajectory through space. Likewise, Wagner (93) described a 66-year-old man who sometimes saw people move very quickly and, at other times, saw them “popping up” in his room or “vanishing” all of a sudden, even though their postures indicated that they must have been walking. Both descriptions show similarities with a case report by Cooper et al. (94) involving a 61-year-old woman who suddenly saw smooth movements “*as a series of discontinuous freeze frames*”; in addition, she saw people in close proximity moving in slow motion. While the latter case was published as an example of akinetopsia and the former two as examples of time distortions, all three papers appear to cover both phenomena.

#### Treatment

Because people experiencing time distortions are hardly ever encountered (or recognized) in clinical practice, evidence-based treatment protocols are non-existent. Therefore, as in other cases of Alice in Wonderland syndrome, practice-based treatments aim to alleviate underlying causes (95). Time distortions in the context of epilepsy are treated with antiepileptics, those in migraine with migraine prophylaxis, propranolol, or antiepileptics, those in encephalitis (depending on the pathogen) with antiviral medications, antibiotics, or immune globulins, those in psychoses with antipsychotics, and so on. In all cases, a proper explanation of the nature and causes of the condition *and* its (suspected) underlying cause are indispensable.

### Future Research

On the basis of our findings, we propose that future research aim at establishing the incidence and prevalence of time distortions in the general population and in specific patient groups. To foster a proper understanding of the outcome of such surveys, the use of a rigorous and well-operationalized classification of time distortions would seem mandatory. In individual cases, we advise to apply neuroimaging and/or other localizing techniques to gain insight into specific neurobiological underpinnings. The interpretation of such findings will obviously stand and fall with neuropsychological testing. As for the suggestion that time distortions may be responsible for triggering cascades of secondary pathology and should therefore perhaps be reconceptualized as core symptoms of disorders such as depression and psychotic disorder, we recommend network analyses to test this hypothesis. Since evidence-based treatment protocols can only be developed with the aid of much larger patient groups than currently available, we have no choice but to wait until such data becomes available. Until then, we can promote awareness of Alice in Wonderland syndrome and its numerous manifestations and urge for its inclusion in major classifications, textbooks, and university courses.

### Limitations

Since the literature on time distortions is rather limited, our analysis was necessarily based on a modest number of cases. Moreover, since reports in the original texts were sometimes incomplete or insufficiently detailed, a robust distinction between time distortions and akinetopsia and related phenomena was not always easy. Even when this distinction was clear, the original studies did not always specify whether the phenomena described therein involved duration evaluation, time expectation, temporal order or asynchrony detection. Also, many of the original studies only allowed us to obtain rather rough indications of the brain regions involved. As a consequence, we were unable to make anatomical claims for neural representations beyond inferences about gross hemispheric or lobar associations derived from the data reviewed. Finally, regarding outcome—which was favorable in two-thirds of the cases—the role of therapeutic interventions was not always clear from the original reports.

## Conclusions

From our systematic analysis of 168 published cases of time distortion characteristic of Alice in Wonderland syndrome we conclude that these discrete, but often intrusive pathological phenomena are experienced at all ages, that they have been reported more often in men than in women, and that, although phenomenologically varied, half of the distortions involve quick-motion and slow-motion phenomena. More than 60% of the cases were multimodal in nature in that there was involvement of the temporal, visual, kinaesthetic, and/or auditory modalities. Neurological disorders were the most prevalent underlying conditions, although type-5 cases (involving bizarre sensations such as time standing still or moving in circles) were mostly seen in the context of psychosis. Overall, mainly occipital areas appear to be involved in the mediation of the time distortions we analyzed, although the temporal network as a whole is taken to be widely disseminated, with separate component timing mechanisms linked to occipital, parietal, temporal, and frontal regions, plus the thalamus and pons. Several rare cases indicate that time distortions may sometimes be accompanied by multimodal desynchronization or even unimodal desynchronization. Even though two-thirds of the cases we analyzed had a favorable prognosis, because of the severity of many of the underlying conditions *and* the 13% death rate we found, we nonetheless advise diagnostics in all cases, including a psychiatric and neurological work-up, blood work, an EEG, a head MRI, and—on indication—further auxiliary investigations. In the absence of evidence-based treatment guidelines we recommend aiming therapeutic interventions at the (suspected) underlying disorder. Scientifically, time distortions offer a unique opportunity to study the nature and neurobiological correlates of lived time, with ramifications for such diverse areas as clinical medicine, neuroscience, psychology, and philosophy. By providing a comprehensive classification of time distortions and related temporal anomalies, we hope to facilitate further research in these disciplines.

## Data Availability Statement

The original contributions presented in the study are included in the article/Supplementary Material, further inquiries can be directed to the corresponding author/s.

## Author Contributions

JDB contributed to the conception and design of the work, and to the analysis and interpretation of data for the work, drafted and revised the work, gave approval for the final version to be published, and agreed to be accountable for all aspects of the work in ensuring that questions related to the accuracy or integrity of any part of the work are appropriately investigated and resolved. NN contributed to the conception and design of the work, and to the acquisition, analysis, and interpretation of data for the work, revised the work, gave approval for the final version to be published, and agreed to be accountable for all aspects of the work in ensuring that questions related to the accuracy or integrity of any part of the work are appropriately investigated and resolved. FW contributed to the drafting and revising of the work, gave approval for the final version to be published, and agreed to be accountable for all aspects of the work in ensuring that questions related to the accuracy or integrity of any part of the work are appropriately investigated and resolved. All authors contributed to the article and approved the submitted version.

## Conflict of Interest

The authors declare that the research was conducted in the absence of any commercial or financial relationships that could be construed as a potential conflict of interest. The reviewer DC declared a past co-authorship with several of the authors JDB, FW to the handling editor.
