# Supplementary material for: Time Distortions: A Systematic Review of Cases Characteristic of Alice in Wonderland Syndrome
Source: Front Psychiatry. 2021 May 7;12:668633. doi: 10.3389/fpsyt.2021.668633 (PMC8138562; doi:10.3389/fpsyt.2021.668633)
Supplement: Supplementary file 1 [file Table_1.docx]

**Supplementary material**

**Table S1** Summary of case descriptions of time distortions (N=84)

| **Case nr.** | **Reference** | **Sex, age**  **(years)** | **Phenomenology** | **Clinical diagnosis** | **Test results** | **Treatment** | **Outcome** |
| --- | --- | --- | --- | --- | --- | --- | --- |
| 1 | Šerko (1913) | M, 33 | **Autodescription: Types 1,2,3:** Temporal: experiencing time to be rushing on, and to lose his grip on time, while things that happened half an hour ago felt like they had happened in a distant past; at the height of intoxication, feeling as if time were limitless (along with metamorphopsias, and complex visual and tactile hallucinations) | Mescaline intoxication | Timing experiment: threefold overestimation of time duration | - | Full recovery in hours |
| 2 | Klien (1919), Pötzl (1939) | M, 8 | **Type 4:** Temporal and visual: quick-motion phenomenon, paroxysmal, for 5 min at a time (seeing people move about rapidly, and hearing them - and himself - speak faster); preceded by the sensation that his whole body becomes thicker | Epilepsy in the context of parotitis, later complicated by dysentery with fever | - | Rest, dietary prescriptions, sodium bromide (used at the time as an antiepileptic and sedative) | Full recovery in weeks |
| 3 | Fischer (1929) | F, - | **Type 5:** Temporal: sensation of time standing still (once) | Schizophrenia | - | - | - |
| 4 | Fischer (1929) | F, ±30 | **Type 5:** Temporal: sensation that time is standing still, that the future is out of reach, and that she is caught in an eternal ‘now’ (permanently) | Schizophrenia | - | - | - |
| 5 | Fischer (1929) | M, - | **Type 5:** Temporal: the recurring sensation that time is going backwards, that the nurses somehow manage to turn time back from 11:30 h to 11:00 h, and that he has to re-experience everything again; plus the sensation that periods in the past get mixed up with each other | Schizophrenia | - | - | - |
| 6 | Fischer (1929) | M, ±40 | **Type 5:** Temporal: sensation that he has been thrown back in time, and that the future and even the present are out of his reach | Schizophrenia | - | - | - |
| 7 | Fischer (1929) | M, - | **Type 5:** Temporal: sensation that time is standing still (recurring), that he is living in an eternal present | Schizophrenia | - | - | - |
| 8 | Lewis (1932) | -,- | **Types 2,3:** Temporal: inability to feel time passing, feeling as if time is standing still (twice) | - | - | - | - |
| 9 | Lewis (1932) | M, - | **Type 3:** Temporal: inability to feel time passing, experiencing an eternal presence | - | - | - | - |
| 10 | Lewis (1932) | M, - | **Type 3:** Temporal: inability to feel time passing, inability to imagine a past and a future | - | - | - | - |
| 11 | Lewis (1932) | - | **Type 5:** Temporal and visual: sensation of time going backwards (lost his sense of the future, and was overwhelmed by memories and images from the past) | - | - | - | - |
| 12 | Lewis (1932) | -,- | **Type 3:** Temporal: sensation of time going fast as well as slow (has the sensation of reading fast, but then notices that hours have gone by) | Oculogyric crisis | - | - | - |
| 13 | Lewis (1932) | F, - | **Types 3,4:** Temporal: quick-motion phenomenon (sees her watch accelerating) and conviction that Greenwich time is much faster than time in the here and now, which appears to go slow | Involutional melancholia | - | - | - |
| 14 | Lewis (1932) | F, - | **Type 5:** Temporal: conviction that the world sometimes stops momentarily, plus the sensation that ‘over here’ it passes more evenly | Schizophrenia | - | - | - |
| 15 | Lewis (1932) | M, - | **Types 2,5:** Temporal and visual: sensation that ‘someone is playing with the clocks’: looks at his watch and looks again an hour later, and it is still the same time; two days ago is like weeks ago; has the sensation of skipping whole days in one night’s sleep | Schizophrenia | - | - |  |
| 16 | Lewis (1932) | M, - | **Type 3:** Temporal: experiencing an eternal presence, without continuity to the past, as if he is reborn every moment and all things are always new | Schizophrenia | - | - | - |
| 17 | Lewis (1932) | M, - | **Type 5:** Temporal and visual: having the sensation of dreaming backwards | Schizophrenia | - | - | - |
| 18 | Hoff & Pötzl (1934) | M, 57 | **Type 4:** Temporal, visual, and auditory: quick-motion phenomenon (seeing physicians in the hospital move as quickly as ‘in a movie that goes too fast’ and hearing music and voices on the left as extremely loud and fast) | Stroke | Lesion to the right occipital lobe with probable involvement of the right parieto-occipital area | - | Full recovery in days |
| 19 | Hoff & Pötzl (1934); Pötzl (1939) | M, 12 | **Type 4:** Temporal, visual, and auditory: quick-motion phenomenon (seeing things move ever faster, and hearing sounds as going ever faster, with an ever higher pitch, until he loses consciousness) | Epileptic aura | - | - | - |
| 20 | Flach & Palisa (1935) | M, 28 | **Types 2,4:** Temporal, visual, and kinesthetic: slow-motion phenomenon (during attacks of oculomotor nerve palsy, feeling himself moving slowly, and seeing people in is surroundings move even slower) plus feeling that time seems to drag | Postencephalitic psychosis with parkinsonism and extrapyramidal symptoms (encephalitis lethargica at age 14) | - | - | Unaltered |
| 21 | Pisk (1936) | F, 45 | **Type 4:** Temporal and visual: during initiation of insulin coma treatment and while waking up, for several minutes, quick-motion phenomenon: seeing people rush past and hearing voices as louder, faster, and higher-pitched; also metamorphopsias (along with preexisting sensed presence and visual, verbal auditory, musical, tactile, somatic, and sexual hallucinations) | Insulin coma treatment in schizophrenia (involution psychosis) | - | Glucose | Full recovery in minutes |
| 22 | Hoff & Pötzl (1937); Pötzl (1939) | M, 62 | **Type 4:** Temporal, visual and auditory: quick- and slow-motion phenomena, rapidly alternating (seeing traffic rushing past and then slowing down, as if almost coming to a halt, apparently going slow in central vision and going fast in peripheral vision); speech sounded normal during slow-motion episodes | Brain infarctions (old and new) | Left-sided hemianopia since 10 years; left-sided hemiplegia a few days before death; PA: left occipital infarction (old) and three new infarctions in the left insula, left planum temporale, and right parietal (supracapsular) region |  | Died in days after new infarctions |
| 23 | Kloos (1938) | F, 30 | **Type 3:** Temporal: inability to experience time, preoccupation with time, ruminating about the past and being unable to picture the future | Depressive disorder | - | - | Recovery in months |
| 24 | Kloos (1938) | F, 51 | **Type 2:** Temporal: Experiencing time to be going slower and almost as standing still | Postmenopausal depression | - | - | Recovery in months |
| 25 | Kloos (1938) | F, 56 | **Type 2:** Temporal: Experiencing time to be going slower (when looking at a clock after completing a task) and almost as standing still | Depressive disorder | - | - | - |
| 26 | Pötzl (1939) | M, 24 | **Type 4:** Temporal and kinesthetic: quick-motion phenomenon (once every 3 weeks on average, having the sensation that his own movements go faster, allowing him to work faster, sometimes leading to ever faster kinesthetic sensations, which are then followed by loss of consciousness) | Epileptic aura | - | - | - |
| 27 | Horányi-Hechst (1943) | F, - | **Types 2,5:** Temporal: after waking up from cardiazol shock treatment, experiencing time as standing still, and then picking up at a very slow pace, with minutes feeling like hours | Cardiazol shock treatment in schizophrenia | - |  | Full recovery in 1 day |
| 28 | Horányi-Hechst (1943) | F, - | **Type 4:** Temporal, visual, and kinesthetic: quick-motion phenomenon (seeing things move faster, and feeling her own movements as slower | Schizophrenia | - |  | - |
| 29 | Pichler (1943) | M, - | **Types 2,4:** Temporal and visual: quick-motion phenomenon, paroxysmal, for hours at a time (seeing people move about rapidly) plus sensation that time passes very slowly | Traumatic right-occipital brain lesion | - | Surgery | Died in 3 weeks due to encephalitis/meningitis |
| 30 | Pichler (1943) and Pötzl (1939) | M, 11 | **Type 4:** Temporal, visual and kinesthetic: quick-motion phenomenon, paroxysmal, for hours at a time (seeing people move about extremely fast and having the sensation that he himself is moving fast, too) | Traumatic right-occipital brain lesion | X-ray: skull perforation | Surgery | Died 7 weeks later due to meningitis |
| 31 | Wagner (1943) | M, 66 | **Type 4:** Temporal, visual and auditory: quick-motion phenomenon and akinetopsia (seeing people move very quickly, and sometimes seeing them ‘popping up’ in his room or vanishing all of a sudden, even though their postures indicate that they are walking) and hearing people speak faster, louder and at a higher pitch | Right parieto-occipital stroke | - | - | - |
| 32 | Bodamer (1947) | M, 36 | **Types 2,4:** Temporal, visual and auditory: quick-motion phenomenon, paroxysmal, for hours at a time (seeing people move about like in old movies, and hearing them speak too fast, in unnatural, high-pitched voices; also the sensation of days, weeks, months flying by) | Traumatic lesion to the right side of the head and blunt trauma to the back of the head | X-ray: superficial shrapnel lesion, skull and brain intact | - | - |
| 33 | Lippman (1951) | F, 29 | **Type 2:** Temporal and kinesthetic: quick-motion phenomenon (having the sensation that she is pulling along at a rapid pace, but seeing herself walking at a normal pace in the reflection of a window) | Migraine | - | - | - |
| 34 | Lippman (1951) | F, 32 | **Type 2:** Temporal and kinesthetic: slow-motion phenomenon (prior to a migraine attack, having the sensation that she is moving very slowly) | Migraine | - | - | - |
| 35 | Pötzl (1951) | M, 32 | **Type 4:** Temporal, visual and auditory: quick-motion phenomenon, paroxysmal (seeing ‘flight lines’ appear that ‘draw him in’, ever faster; seeing people and objects move too fast; also hearing sounds too fast, without any changes in pitch); is sometimes able to abort the sensation by repeatedly looking to objects on the left and right | Epileptic aura | - | - | - |
| 36 | Becker & Sternbach (1953) | M, 51 | **Types 2,3:** Temporal: experiencing epileptic seizures and the sensation that time is rushing past (the past years feel like months, months seem like a single day) | Left thalamic stroke with hemiplegia and secondary epilepsy | EEG: slow waves over the left occipital lobe | - | - |
| 37 | Becker & Sternbach (1953) | M, 51 | **Types 2,4:** Temporal and visual: experiencing the days as extremely long, as if time never passes; plus central quick-motion phenomenon (seeing people and objects moving extremely fast in the central field of vision, and normally in the periphery) | Left thalamic stroke with hemiplegia and secondary epilepsy | - | Periarterial injection of the left carotid artery with Novocain | Full recovery after injection |
| 38 | Critchley (1953) | M, - | **Type 3:** Temporal: inability to feel time passing | Right fronto-parietotemporal astrocytoma (unverified) | - | - | - |
| 39 | Häfner (1953) | M, 54 | **Type 3:** Temporal: inability to feel time passing (along with apathy and frontal-lobe syndrome) | Shrapnel wound to the right frontal lobe, secondary epilepsy | - | Surgery | Unaltered |
| 40 | Häfner (1953) | M, 10 | **Type 3:** Temporal: inability to feel time passing (along with constant agitation, and regressive and impulsive behaviour) | Shrapnel wound to the head | X-ray: shrapnel located over the midline and in the left angulus marginalis | Conservative treatment | Unaltered |
| 41 | Häfner (1953) | M, 48 | **Types 2,4:** Temporal and visual: quick-motion and slow-motion phenomena, paroxysmal, for minutes at a time (seeing people move about very slowly or very rapidly, especially when he changes his own speed) plus sensation that time passes very slowly | Traumatic postcontusional syndrome with insomnia and prosopagnosia | X-ray: shrapnel near the right occipital lobe, probably extracerebral; arteriogram: caliber changes of the calloso-marginal artery, left and right | - | - |
| 42 | Häfner (1953) | M, 41 | **Type 4:** Temporal, visual and kinesthetic: quick-motion phenomenon, paroxysmal, for 15-60 min at a time (seeing people move about extremely fast and having the sensation that he himself is moving fast, too) while experiencing himself as very slow | Status after skull-base fracture due to blunt head trauma | Pneumencephalography: enlargement of third ventricle; EEG: normal | - | - |
| 43 | Lippman (1953) | F, 36 | **Type 3:** Temporal: during an out-of-body experience, having the sensation of being two persons, being suspended up above her physical body, and experiencing time as ceasing to exist | Migraine | - | - | Full recovery in minutes (though recurring) |
| 44 | Gloning & Weingarten (1954) | M, 49 | **Type 4:** Temporal, visual, auditory, and kinesthetic: quick-motion phenomenon (seeing things going faster (only in the peripheral field of vision), hearing sounds as faster, with a higher pitch, and having the feeling (during attacks of 1 hour) to be moving very fast, ‘as if on roller skates’) | Brain tumor | EEG: diffuse beta waves; pneumencephalography, arteriography, and phlebogram: signs of hydrocephalus and thalamic tumor | - | - |
| 45 | Todd (1955) | F, 32 | **Type 2 or type 4:** Temporal: slow-motion phenomenon (illusory slowing of the passage of time during paroxysmal episodes of Alice in Wonderland syndrome) | Alice in Wonderland syndrome in migraine or epilepsy | EEG: generalised dysrhythmia with paroxysmal disturbances in both anterior temporal areas, but no epileptic discharges | - | - |
| 46 | Efron (1956) | F, ±50 | **Type 4:** Temporal and kinesthetic: slow-motion phenomenon, paroxysmal (experiencing herself as very slow, for 10-30 minutes at a time, while doing things ‘at breakneck speed’) | Epileptic aura | - | - | - |
| 47 | Mullan & Penfield (1959) | F, 27 | **Type 4:** Temporal and visual: slow-motion phenomenon | Epilepsy secondary to brain tumor | PA: right temporal glioma | Surgery | - |
| 48 | Cohen (1966) | M, - | **Type 5:** Temporal: time would completely stop, then start again | HPPD after LSD use | - | phenothiazine | Partial recovery in 5 weeks |
| 49 | Arieti (1974) | F, 29 | **Type 3:** Temporal: inability to feel time passing | Postpartum psychosis | - | Psychotherapy | - |
| 50 | Golden (1979) | F, 11 | **Type 4:** Temporal and visual: quick-motion phenomenon: seeing things moving too fast | Alice in Wonderland syndrome in migraine | EEG: normal | - | - |
| 51 | Golden (1979) | M, 11 | **Type 4:** Temporal, visual and auditory: quick-motion phenomenon: seeing things moving too fast, and hearing people talking too fast | Alice in Wonderland syndrome in migraine | EEG: normal (twice) | - | - |
| 52 | Pethö (1985) | F, 25 | **Types 2,5:** Temporal: reduplication of events in time (is convinced that she has experienced everything before) and déjà vécu | Psychotic disorder | EEG, CSF, and pneumencephalography: normal | Antipsychotics, ECT | Unaltered for 12 years |
| 53 | Levi & Miller (1990) | M, 25 | **Types 1,4:** Temporal and visual: quick-motion phenomenon: seeing things speed up, as if he were ‘watching a motion picture at fast speed’; later also confusion about temporal events | HPPD after use of marijuana, cocaine and possibly a hallucinogenic | EEG, CT, MRI, ophthalmology, and toxicology normal | Psychotherapy, tricyclic antidepressants, carbamazepine, propranolol | Unaltered in 6 years |
| 54 | Binkofski & Block (1996) | M, 66 | **Types 2,4**: Temporal and visual: quick-motion phenomenon (suddenly perceiving oncoming traffic as excessively fast, and TV programmes as too quick) plus the feeling that life passes very quickly | Brain tumor | CT: left prefrontal cystic lesion; EEG: high-amplitude delta activity over left frontal areas (no epileptic activity); PA: high-grade glioblastoma | - | - |
| 55 | Mizuno et al. (1998) | M, 54 | **Type 2:** Temporal and visual: inability to assess time correctly, having the sensation that the days pass extremely quickly and that past events all happened at the same time; inability to estimate the speed of cars | Depressive disorder with Alice in Wonderland syndrome and attempted suicide (twice) | CSF, CT, MRI, EEG, and neuropsychological testing: normal | clomipramine | Full recovery in 2 days; lost to follow-up after 6 months |
| 56 | Pérez Méndez et al. (2001) | M, 6 | **Type 2:** Temporal and auditory: hearing his mother speak too fast (along with metamorphopsias) | Alice in Wonderland syndrome in EBV infection | EEG: normal; EBV serology positive | - | Full recovery in 2 days |
| 57 | Takaoka et al. (2001) | F, 22 | **Type 4**: Temporal and visual: slow-motion phenomenon (‘Everything is moving as in a slow-motion picture’) | Alice in Wonderland syndrome in toluene-based brain dysfunction | EEG: normal; CT: slight atrophy of the cortex | - | Full recovery in 6 months |
| 58 | Giannotti (2003) | F, 9 | **Type 2:** Temporal and auditory: on 3 occasions, hearing people speak too fast (along with metamorphopsias) | Alice in wonderland syndrome in infectious mononucleosis (Epstein Barr) | EBV serology positive | - | Full recovery in 2 months |
| 59 | Kitchener (2004) | M, 6 | **Type 4:** Temporal and kinesthetic: quick-motion phenomenon (feeling that he were moving too fast) | Alice in Wonderland syndrome in typhoid encephalopathy | CT and EEG: normal; MRI: right temporo-occipital edema | amoxicillin, prednisolone | Full recovery in 3 weeks |
| 60 | Hamilton et al. (2006) | M, 53 | **Type 4:** Temporal: unspecified temporal dissociation between auditory and visual input (seeing people’s lips move out of sync with the words they speak) | Possible right ischemic event (DD: TIA, migrainous infarct) | MRI: normal; SPECT: biparietal hypoperfusion, right possibly more than left | - | - |
| 61 | Augarten & Aderka (2011) | F, 11 | **Type 4**: Temporal and visual: slow-motion phenomenon (during a bout of fever, seeing her parents move in slow motion and hearing them speak very slowly) | Alice in Wonderland syndrome in H1N1 influenza | EBV serology negative; polymerase chain reaction for H1N1 positive | oseltamivir phosphate | Full recovery in 2 days |
| 62 | Weidenfeld & Borusiak (2011) | M, 9 | **Type 4:** Temporal, visual, and auditory: quick-motion phenomenon (for 5-10 minutes at a time, seeing everything as going very fast, and hearing everything as very fast, ‘like you put an old-fashioned record player on 45 rotations per minute instead of 33’) | Alice in Wonderland syndrome | EEG and CSF: normal | - | - |
| 63 | Cooper et al. (2012) | F, 61 | **Type 4**: Temporal and visual: slow-motion phenomenon (suddenly experiencing smooth movements of people as ‘a series of freeze frames’ (akinetopsia) and seeing people close by move very slowly) | Brain infarction | MRI and DWI: multiple old and new infarctions in the cortex and subcortical white matter of the right inferior parietal lobe and right parieto-occipital junction (none on the left) | - | Full recovery in 5 days |
| 64 | Wanigasinghe (2012) | F, 12 | **Type 4:** Temporal and kinesthetic: quick-motion phenomenon (feeling of her actions ‘speeding up’) | Alice in Wonderland syndrome in migraine | EEG: normal; EBV serology: negative | carbamazepine, migraine prophylaxis | Partial recovery in 2 months |
| 65 | Freeman et al. (2013) | M, 67 | **Type 4:** Temporal, visual and kinesthetic: slow-motion phenomenon (seeing people’s lips move out of sync with the words they speak, and hearing his own spoken words before feeling his own mouth movements) | Brain infarction | MRI: lacunar infarcts in the left subthalamic nucleus and in the right pontine nucleus; DTI: both lesions implicated in cross-modal interactions | - | - |
| 66 | George & Bernard (2013) | F, 13 | **Type 2:** Temporal: inability to assess time correctly, having the sensation that time goes extremely slowly | Migraine and panic attacks with hallucinations | MRI and EEG: normal | pitozifen, zolmitriptan, psychotherapy | Full recovery in a month |
| 67 | Ovsiew (2013) | M, 39 | **Type 4:** Temporal and visual: slow-motion phenomenon (suddenly perceiving droplets under the shower as suspended in mid-air, ‘very similar to the way the bullets travelled in the Matrix movies’) | Brain hematoma | CT and MRI: large right temporal hematoma plus small subdural hematoma; MRA: bleeding from a right temporal arteriovenous malformation plus unruptured carotid aneurysms | Surgery (one month later) and clipping of the AVM; antiepileptics for subsequent seizures (clouding of consciousness) | Full recovery in minutes |
| 68 | Ilik & Ilik (2014) | M, 14 | **Type 4:** Temporal and kinesthetic: quick-motion phenomenon (feeling that he moved very fast) | Alice in Wonderland syndrome as migraine aura | MRI and EEG: normal | propranolol, sodium valproate | Full recovery within 1 year while on valproic acid |
| 69 | Lerner et al. (2014) | M, 26 | **Type 4:** Temporal and visual: slow-motion phenomenon (seeing things move slowly) | HPPD after cannabis use | EEG: normal | clonazepam | Substantia recovery in 6 months |
| 70 | Brodrick & Mitchell (2015) | M, 30 | **Type 4:** Temporal and visual: quick-motion phenomenon (seeing people move faster than normal, continuously) | HPPD (after use of cannabis laced with LSD and phencyclidine, as well as cocaine) and bipolar disorder | MRI: nonspecific findings of scattered foci | citalopram, lamotrigine, mirtazapine, levetiracetam, and other psychotropics | Death after completed suicide, 1 week after transfer to another hospital |
| 71 | Uca & Kozak (2015) | M, 35 | **Type 4:** Temporal and kinesthetic: slow-motion phenomenon (‘a slowing down of overall movements’) | Alice in Wonderland syndrome in cluster headache | MRI and EEG: normal | sumatriptan, methylprednisolone, valproic acid | Full recovery in a month after addition of valproate |
| 72 | Perdices (2016) | F, 45 | **Type 2 or type 5:** Temporal: on 3 occasions, having the feeling that time were standing still | Alice in Wonderland syndrome | CT, MRI and EEG: normal; neuropsychological assessment: mild deficits in attention, memory and executive functions | - | - |
| 73 | Almeida & Valença (2017) | F, 32 | **Type 4:** Temporal and visual: slow-motion phenomenon (at two occasions, experiencing herself as moving faster, and her surroundings as if in slow motion) | Alice in Wonderland syndrome in migraine | MRI and MRA: normal | Migraine medication, topiramate | Full recovery within 30 min) |
| 74 | Yokoyama et al. (2017) | M, 63 | **Type 2:** Temporal: inability to assess time correctly, having the sensation that the days pass extremely quickly | Depressive disorder with Alice in Wonderland syndrome (2 episodes) | MRI and EEG: normal; FDG-PET: hypermetabolism frontal cortex, decreased after treatment | First episode: amitriptyline, perphenazine; second episode: duloxetine, mirtazapine, and ECT (12 sessions) | Full recovery in months |
| 75 | Beh et al. (2018) | F, 39 | **Type 4:** Temporal and visual: slow-motion phenomenon (while driving, experiencing everything as moving in slow motion) | Alice in Wonderland syndrome in vestibular migraine | MRI and EEG: normal | Antimigraine medication, nortriptyline | Full recovery in 5 minutes |
| 76 | Funayama et al. (2018) | F, 31 | **Type 5:** Temporal and visual (while recovering from surgery, having the sensation that time progressed backwards while looking at a traditional clock on the wall) | Alice in Wonderland syndrome during postoperative state in ANMDAR encephalitis with teratomata and intractable seizures | MRI: normal; EEG: slowing of the alpha wave; CSF: antibodies against NMDAR | Mechanical ventilation, surgery (teratomectomy) and methylprednisolone | Full recovery in 3 months |
| 77 | Funayama et al. (2018) | F, 29 | **Types 2,4:** Temporal, visual, and auditory: slow-motion phenomenon (feeling that the day is 48 h long, seeing people move slowly, hearing them laugh and speak slowly, and hearing music as too slow) | Alice in Wonderland syndrome in ANMDAR encephalitis with teratomata and intractable seizures | MRI and EEG: normal; CSF: antibodies against NMDAR | Mechanical ventilation, surgery (teratomectomy), methylprednisolone, immunoglobulins, plasma exchanges | - |
| 78 | Jia et al. (2018) | M, ±28 | **Type 4:** Temporal, visual, and auditory: quick-motion phenomenon (since childhood, experiencing paroxysmal attacks during which things appear to go faster, and people speak too fast) | Alice in Wonderland syndrome in migraine | Reaction time experiments: over a third slower during episodes of AIWS | - | - |
| 79 | Martin et al. (2018) | M, 22 | **Type 3:** Temporal: inability to feel time passing | Schizophrenia | Neuropsychological assessment: timing disorder | - | - |
| 80 | Matsuura et al. (2019) | F, 8 | **Type?** Temporal: unspecified ‘altered experience of psychological time (time distortion)’ | Alice in Wonderland syndrome in migraine | MRI and EEG: normal | lomerizine, valproate sodium | Full recovery of time distortions after start with lomerizine |
| 81 | Naarden et al. (2019) | M, 68 | **Type 4:** Temporal and visual: quick- and slow-motion phenomena (‘like in a Charlie Chaplin movie’) | Alice in Wonderland syndrome in Creutzfeldt-Jakob disease | MRI: cortical ribbon sign in left parieto-occipital cortex; EEG: periodic sharp-wave complexes over the same area; CSF: 14-3-3 proteins and elevated tau protein | - | Died in 1 month |
| 82 | Blom (2020) | M, 50 | **Type 3:** Temporal: inability to feel time passing | Cannabis intoxication | - | - | Full recovery in days |
| 83 | Blom (2020) | M, 28 | **Type 4:** Temporal and visual: quick- and slow-motion phenomena, paroxysmal and rapidly alternating (seeing traffic rushing past and then slowing down, as if almost coming to a halt) | Schizophrenia | - | risperidone | - |
| 84 | Kesserwani (2020) | M, 15 | **Type 4:** Temporal, visual and possibly kinesthetic: slow-motion phenomenon (experiencing all things as going slower; the left arm and leg either moving slower or being ataxic); along with blurring of vision in the left hemifield | Cryptogenic ischemic brain infarct | MRI, FLAIR MRI, and gradient echo: infarction of the right-sided banks of the parieto-occipital area; CTA, duplex of the carotids, CSF, and ECG monitoring: normal; thrombophilia panel: factor V Leiden mutation |  | Full recovery in 12 hours |

**References**

Almeida, L.C.A., and Valença, M.M. (2017). “Um mundo em cȃmera lenta” como manifestaçao da Síndrome de Alice no País das Maravilhas: Um relato de caso. *Headache Medicine*. 8, 134-137.

Arieti, S. (1974). *Interpretation of Schizophrenia. Second Edition, Completely Revised and Expanded*. New York, NY: Basic Books.

Augarten, A., and Aderka, D. (2011). Alice in Wonderland syndrome in H1N1 influenza. *Pediatr. Emerg. Care*. 27, 120.

Becker, A.M., and Sternbach, I. (1953). Über Zeitsinnstörung bei Thalamusherden. *Wien. Z. Nervenheilkd*. 7, 62-67.

Beh, S.C., Masrour, S., Smith, S.V., Friedman, D.I. (2018). Clinical characteristics of Alice in Wonderland syndrome in a cohort with vestibular migraine. *Neurol. Clin. Pract.* 8, 389-396.

Binkofski, F., and Block, R.A. (1996). Accelerated time experience after left frontal cortex lesion. *Neurocase*. 2, 485-493.

Blom, J.D. (2020). *Alice in Wonderland Syndrome*. Cham: Springer Nature.

Bodamer, J. (1947). Die Prosop-Agnosie (die Agnosie des Physiognomieerkennens). *Arch. Psychiatr. Nervenkr.* 179, 6-53.

Brodrick, J., and Mitchell, B.G. (2015). Hallucinogen persisting perception disorder and risk of suicide. *J. Pharm. Pract*. 29, 431-434.

Cohen, S. (1966). A classification of LSD complications. *Psychosomatics*. 7, 182-186.

Cooper, S.A., Joshi, A.C., Seenan, P.J., Hadley, D.M., Muir, K.W., Leigh, R.J., et al. (2012). Akinetopsia: Acute presentation and evidence for persisting defects in motion vision. *J. Neurol. Neurosurg. Psychiatry*. 83, 228-229.

Critchley, M. (1953). *The Parietal Lobes*. London: Edward Arnold & Co, 353.

Efron, R. (1956). The effect of olfactory stimuli in arresting uncinate fits. *Brain*. 79, 267-281.

Fischer, F. (1929). Zeitstruktur und Schizophrenie. *Z. Gesamte. Neurol. Psychiatr.* 121, 544-574.

Flach, A., and Palisa, C. (1935). Zur Psychopathologie des Zeiterlebens im postencephalitischen Blickkrampf. *Z. Gesamte. Neurol. Psychiatr.* 154, 599-620.

Freeman, E.D., Ipser, A., Palmbaha, A., Paunoiu, D., Brown, P., Lambert, C., et al. (2013). Sight and sound out of synch: Fragmentation and renormalisation of audiovisual integration and subjective timing. *Cortex*. 49, 2875-2887.

Funayama, M., Mizushima, J., Takata, T., Koreki, A., Mimura, M. (2018). Altered perception might be a symptom of anti-N-methyl-D-aspartate receptor (NMDAR) encephalitis. *Neurocase*. 24, 255-258.

George, D., and Bernard, P. (2013). Complex hallucinations and panic attacks in a 13-year-old with migraines: The Alice in Wonderland syndrome. *Innov. Clin. Neurosci.* 10, 30-32.

Giannotti, A.M. (2003). Síndrome de “Alicia en el País de las Maravillas” e infección por virus de Epstein Barr. *Arch. Argent. Pediatr*. 101, 41-43.

Gloning, I., and Weingarten, K. (1954). Über einen Fall mit taktilem und kinästethischem Zeitraffer. *Wien. Z. Nervenheilkd. Grenzgeb*. 8, 237-241.

Golden, G.S. (1979). The Alice in Wonderland syndrome in juvenile migraine. *Pediatrics*. 63, 517-519.

Häfner, H. (1953). Psychopathologie der cerebralorganisch bedingten Zeitsinnestörungen. *Archiv für Psychiatrie und Zeitschrift Neurologie*. 190, 530-545.

Hamilton, R.H., Shenton, J.T., Coslett, H.B. (2006). An acquired deficit of audiovisual speech processing. *Brain. Lang.* 98, 66-73.

Hoff, H., and Pötzl, O. (1934). Über eine Zeitrafferwirkung bei homonymer linksseitiger Hemianopsie. *Z. Gesamte. Neurol. Psychiatr.* 151, 599-641.

Hoff, H., and Pötzl, O. (1937). Anatomischer Befund eines Falles mit Zeitrafferphänomen. *Dtsch. Z. Nervenheilkd.* 145**,**150-178.

Horányi-Hechst, B. (1943). Zeitbewußtsein und Schizophrenie. *Archiv für Psychiatrie*. 116, 287-292.

Ilik, F., and Ilik, K. (2014). Alice in Wonderland syndrome as aura of migraine. *Neurocase*. 20, 474-475.

Jia, Y., and Miao, Y. (2018). Evidence for the perception of time distortion during episodes of Alice in wonderland syndrome. *J. Nerv. Ment. Dis.* 206, 473-475.

Kesserwani, H. (2020). The Zeitraffer phenomenon: A strategic ischemic infarct of the banks of the parieto-occipital sulcus - A unique case report and a side note on the neuroanatomy of visual perception. *Cureus*. 12, e9443.

Kitchener, N. (2004). Alice in Wonderland syndrome. *International Journal of Child Neuropsychiatry*. 1, 107-112.

Klien, H. (1919). Beitrag zur Psychopathologie und Psychologie des Zeitsinns. *Zeitschrift für Pathopsychologie*. 3, 307-362.

Kloos, G. (1938). Störungen des Zeiterlebens in der endogenen Depression. *Nervenarzt*. 11, 225-244.

Lerner, A.G., Goodman, C., Bor, O., Rudinski, D., Lev-Ran, S. (2014). Synthetic cannabis substances (SPS) use and hallucinogen persisting percerption disorder (HPPD): Two case reports. *Isr. J. Psychiatry. Relat. Sci*. 51, 277-280.

Levi, L., and Miller, N.R. (1990). Visual illusions associated with previous drug abuse. *J. Clin. Neuroopthalmol*. 10, 103-110.

Lewis, A. (1932). The experience of time in mental disorder. *J. R. Soc. Med*. 25, 611-620.

Lippman, C.W. (1951). Hallucinations in migraine. *Am. J. Psychiatry*. 107, 856-858.

Lippman, C.W. (1953). Hallucinations of physical duality in migraine. *J. Nerv. Ment. Dis*. 117, 345-350.

Martin, B., Franck, N., Cermolacce, M., Coull, J.T., Giersch, A. (2018). Minimal self and timing disorders in schizophrenia: A case report. *Front. Hum. Neurosci*. 12, 132.

Matsuura, K., Ishikura, R., Oguri, M., Saito, Y. (2019). Atypical symptoms in migraine-related Alice in Wonderland syndrome: Expansion of the phenotype and reflection on the pathomechanism. *Yanoga. Acta. Med*. 62, 163-165.

Mizuno, M., Kashima, H., Chiba, H., Murakami, M., Asai, M. (1998). ‘Alice in Wonderland’ syndrome as a precursor of depressive disorder. *Psychopathology.* 31, 85-89.

Mullan, S., and Penfield, W. (1959). Illusions of comparative interpretation and emotion: Production by epileptic discharge and by electrical stimulation in the temporal cortex. *AMA. Arch. Neurol. Psychiatry*. 81, 269-284.

Naarden, T., ter Meulen, B.C., van der Weele, S.I., Blom, J.D. (2019). Alice in wonderland syndrome as a presenting manifestation of Creutzfeldt-Jakob disease. *Front. Neurol.* 10, 1-6.

Ovsiew, F. (2013). The *Zeitraffer* phenomenon, akinetopsia, and the visual percpetion of speed of motion: A case report. *Neurocase*. 20, 269-272.

Perdices, M. (2018). The Alice in Worderland syndrome. *Neuropsychol. Rehabil.* 28, 189-198.

Pérez Méndez, C., Martín Mardomingo, M.A., Otero Martínez, B., Lagunilla Herrero, L., Fernández Zurita, C. (2001). Síndrome de “Alicia en el País de las Maravillas” asociado a infección por el virus de Epstein-Barr. An. Esp. Pediatr. 54, 601-602.

Pethö, B. (1985). Chronophrenia - A new syndrome in functional psychoses. *Psychopathology*. 18, 174-180.

Pichler, E. (1943). Über Störungen des Raum- und Zeiterlebens bei Verletzungen des Hinterhauptlappens. *Z. Gesamte. Neurol. Psychiatr.* 176, 434-464.

Pisk, G. (1936). Über ein “Zeitraffer“phänomen nach Insulinkoma. Z. Gesamte. Neurol. Psychiatr. 156**,**777-786.

Pötzl, O. (1939). Physiologisches und Pathologisches über das persönliche Tempo. *Wien. Klin. Wochenschr*. 52, 569-573.

Pötzl, O. (1951). Weiteres über das Zeitraffer-Erlebnis. *Wien. Z. Nervenheilkd. Grenzgeb*. 4, 9-39.

Šerko, A. (1913). Im Mescalinrausch. *Jahrbücher für Neurologie und Psychiatrie* 34, 355-366.

Takaoka, K., Ikawa, N., Niwa, N. (2001). ‘Alice in Wonderland’ syndrome as a precursor of delusional misidentification syndromes. *Int. J. Psychiatry. Clin. Pract*. 5, 149-151.

Todd, J. (1955). The syndrome of Alice in Wonderland. *Can. Med. Assoc. J*. 73, 701-704.

Uca, A.U., and Kozak, H.H. (2015). The Alice in Wonderland syndrome: A case of aura accompanying cluster headache. *Balkan. Med. J*. 32, 320-322.

Wagner, W. (1943). Anisognosie, Zeitrafferphänomen und Uhrzeitagnosie als Symptome der Störungen im rechten Parieto-Occipitallappen. *Nervenarzt*. 16, 49-57.

Wanigasinghe, J. (2012). Alice in Wonderland syndrome: A migraine variant. *Sri Lanka Journal of Child Health*. 41, 40-41.

Weidenfeld, A., and Borusiak, P. (2011). Alice-in-Wonderland syndrome--a case-based update and long-term outcome in nine children. *Childs. Nerv. Syst*. 27, 893-896.

Yokoyama, T., Okamura, T., Takahashi, M., Momose, T., Kondo, S. (2017). A case of recurrent depressive disorder presenting with Alice in Wonderland syndrome: Psychopathology and pre- and post-treatment FDG-PET findings. *BMC. Psychiatry*. 17, 4-9.
